# Microorganisms associated with hedgehog arthropods

**DOI:** 10.1186/s13071-023-05764-7

**Published:** 2023-06-22

**Authors:** Linda Benkacimi, Adama Zan Diarra, Jean-Michel Bompar, Jean-Michel Bérenger, Philippe Parola

**Affiliations:** 1 Aix Marseille Univ, IRD, AP-HM, SSA, Vecteurs–Infections Tropicales et Méditeranéennes (VITROME), Marseille, France; 2 IHU-Méditerranée infection, Marseille, France; 3 Société Française d’Études et de Protection des Mammifères (SFEPM), Bourges, France

**Keywords:** Hedgehogs, Ticks, Fleas, Microorganisms, Zoonoses

## Abstract

Hedgehogs are small synanthropic mammals that live in rural areas as well as in urban and suburban areas. They can be reservoirs of several microorganisms, including certain pathogenic agents that cause human and animal public health issues. Hedgehogs are often parasitized by blood-sucking arthropods, mainly hard ticks and fleas, which in turn can also carry various vector-born microorganisms of zoonotic importance. Many biotic factors, such as urbanization and agricultural mechanization, have resulted in the destruction of the hedgehog’s natural habitats, leading these animals to take refuge near human dwellings, seeking food and shelter in parks and gardens and exposing humans to zoonotic agents that can be transmitted either directly by them or indirectly by their ectoparasites. In this review, we focus on the microorganisms detected in arthropods sampled from hedgehogs worldwide. Several microorganisms have been reported in ticks collected from these animals, including various *Borrelia* spp*., Anaplasma* spp*., Ehrlichia* spp*.*, and *Rickettsia* spp. species as well as *Coxiella burnetii* and *Leptospira* spp. As for fleas, *C. burnetii*, *Rickettsia* spp., *Wolbachia* spp., *Mycobacterium* spp. and various *Bartonella* species have been reported. The detection of these microorganisms in arthropods does not necessarily mean that they can be transmitted to humans and animals. While the vector capacity and competence of fleas and ticks for some of these microorganisms has been proven, in other cases the microorganisms may have simply been ingested with blood taken from an infected host. Further investigations are needed to clarify this issue. As hedgehogs are protected animals, handling them is highly regulated, making it difficult to conduct epidemiological studies on them. Their ectoparasites represent a very interesting source of information on microorganisms circulating in populations of these animals, especially vector-born ones.

**Graphical abstract:**

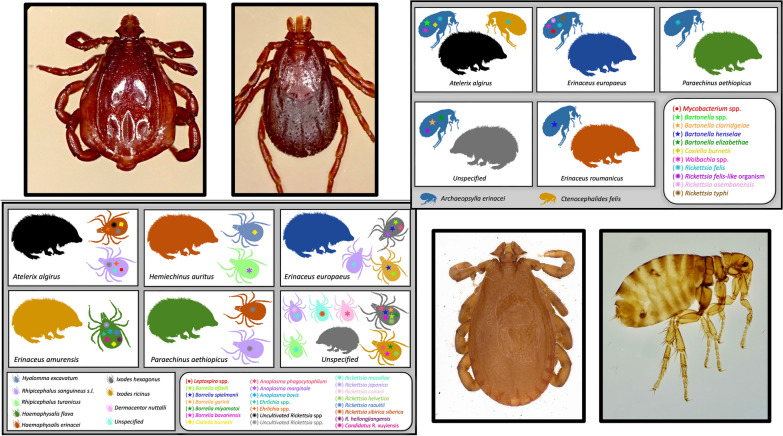

## Background

Hedgehogs are small synanthropic nocturnal omnivorous mammals whose dorsal part of the skin is wholly covered with spines [1, 2]. They are hibernating animals, dig their own burrows, and live in rural areas as well as in urban and suburban areas [1]. Hedgehogs belong to the Erinaceidae family, and include up to 17 known species divided into five genera (*Erinaceus*, *Atelerix*, *Hemiechinus*, *Paraechinus* and *Mesechinus*) distributed worldwide [3, 4]. Hedgehogs are found in Europe, Africa and Asia and were introduced by importation into New Zealand and the Americas [1, 4, 5]. Hedgehog populations have been continuously decreasing, leading the authorities of several countries to provide protection by classifying them in their lists of species threatened with extinction. It is therefore prohibited to kill them or to retain them in captivity [3].

Interest in these animals has increased over the years. The first studies, dating from the late nineteenth century, were mainly focused on the histology, physiology and anatomy of hedgehogs [6]. Researchers have been very interested in their hibernation process, mainly in the physiological changes allowing them to go from an homeothermic animal status to a poikilothermic animal status and vice versa [7].

In the 1940s, laboratory-reared populations of *Erinaceus europaeus* hedgehogs were established as a means to meet research needs [8]. However, it was only in the 1950s that researchers began to show greater interest in their role as reservoirs for pathogens involved in human and animal public health issues and possibly responsible for zoonoses [5, 9, 10]. Laboratory-reared hedgehog specimens were used in experimental models which aimed to determine their sensitivity to different microorganisms, including *Borrelia* strains (*B. duttoni*, *B. crocidurae*, *B. hispanica* and *B. persica*) [9]. The experimental infection of a hedgehog by *Trypanosoma gambiense* showed that this parasite caused a chronic infection in the animal leading to its death [11]. Different microorganisms have already been reported in hedgehogs, including bacteria (*Yersinia pseudotuberculosis*, *Coxiella burnetii*, *Mycobacterium* spp., *Corynebacterium* spp., methicillin-resistant *Staphylococcus aureus*, *Salmonella* spp., *Leptospira* spp., *Chlamydia psittaci* and *Streptococcus* spp.), protozoa (*Cryptosporidium* spp., *Toxoplasma gondii*), fungus (*Candida albicans*, *Microsporum* spp., *Trichophyton erinacei*) and viruses (Coronaviruses, *Herpesvirus*, rabies virus, *Paramyxovirus*) [2, 3]. Similarly, several vector-borne microorganisms have also been detected, including *Tick-borne encephalitis virus* (TBEV), *Crimean-Congo hemorrhagic fever orthonairovirus* (CCHFV), Severe Fever with Thrombocytopenia Syndrome Virus (SFTSV), *Bhanja bandavirus*, *Yersinia pestis*, *Rickettsia* spp., *Bartonella* spp., *Babesia* spp., *Candidatus* Neoehrlichia mikurensis and *Leishmania* spp. [2, 3, 12–15]. In addition, several studies have reported hedgehog infection by *Borrelia burgdorferi* sensu stricto (*B. burgdorferi* s.s.)*,* the agent of Lyme disease [3, 16], and *Anaplasma phagocytophilum*, the agent of granulocytic anaplasmosis [3, 12].

**Fig. 1:**
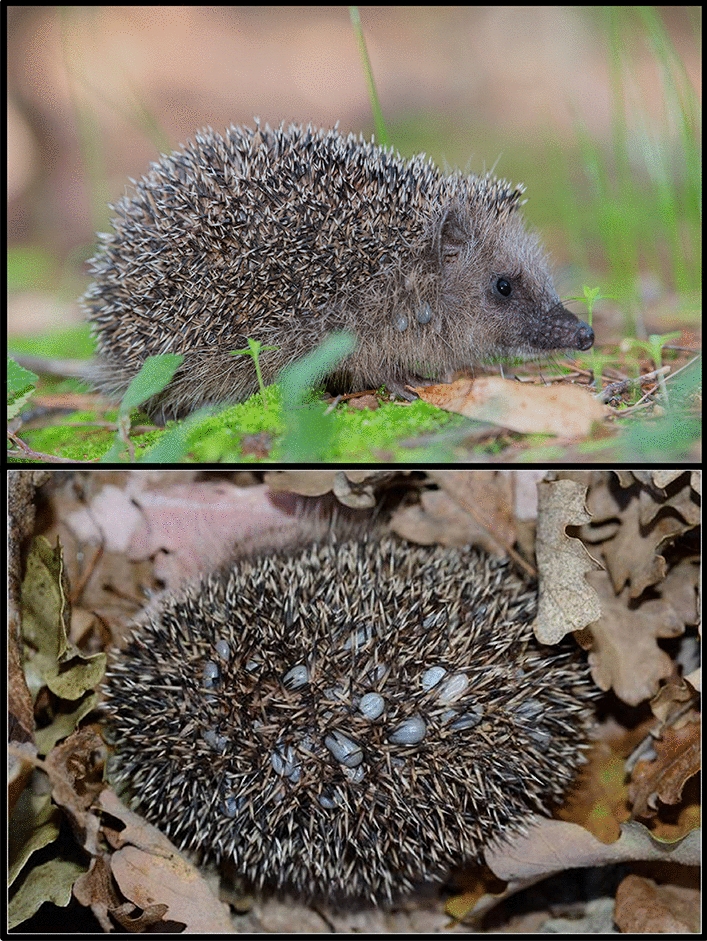
Hedgehogs parasitized by ticks

**Fig. 2:**
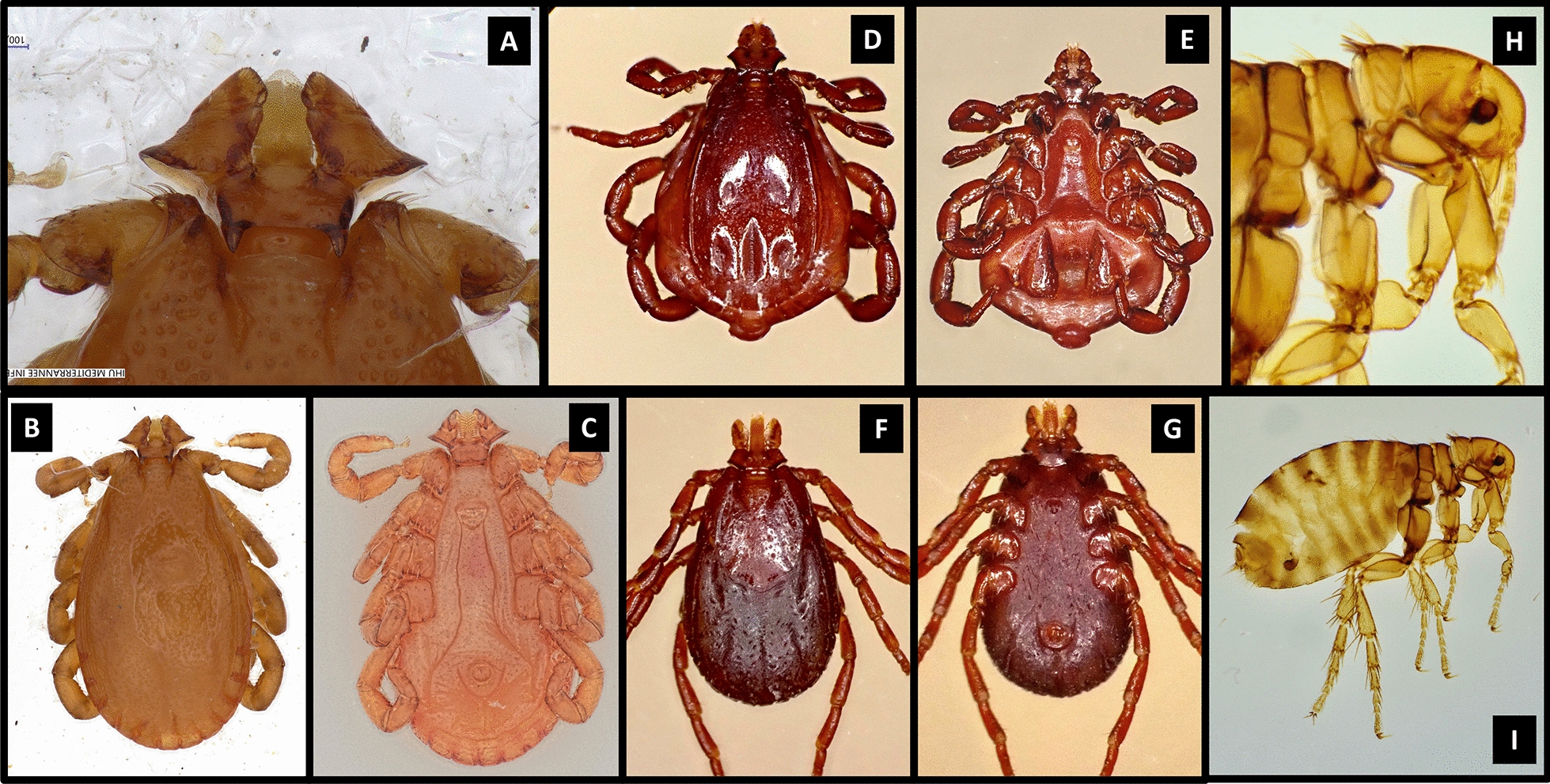
Photographs of arthropods sampled from *Atelerix algirus* hedgehogs taken with a Zeiss Axio Zoom V16 stereomicroscope (Carl Zeiss Meditec France S.A.S., Marly le Roi, France) and a VHX-7000 digital microscope (Keyence Corp., Osaka, Japan). **a**–**c** *Haemaphysalis erinacei* capitulum, dorsal and ventral views, **d**, **e** *Rhipicephalus sanguineus* sensu lato male dorsal and ventral views, **f**, **g** *Rhipicephalus sanguineus* sensu lato female dorsal and ventral views,** h**,** i** *Archaeopsylla erinacei* female flea

Hedgehogs are often parasitized by mites and blood-sucking arthropods (Fig. 1), mainly hard ticks and fleas (Fig. 2) [12, 13].* Ixodes hexagonus* and* Ixodes ricinus* are the most frequently collected ticks from hedgehogs [3, 13, 17, 18], but other tick species have also been reported, including* Rhipicephalus sanguineus* sensu lato (*Rh*.* sanguineus* s.l.) (Fig. 2), *Rhipicephalus* (*Boophilus*) *annulatus*,* Rhipicephalus camicasi*,* Haemaphysalis erinace*i (Fig. 2), *Haemaphysalis flava*, *Haemaphysalis longicornis*, *Haemaphysalis colesbergensis*,* Haemaphysalis norvali*, *Amblyomma marmoreum*,* Dermacentor nuttalli*, *Hyalomma aegyptium*, *Hyalomma excavatum*, *Hyalomma asiaticum*, *Hyalomma dromedarii*, *Hyalomma schulzei*,* Ixodes acuminatus*, *Rhipicentor nuttalli* and the soft tick *Ornithodoros* [4, 13, 16–30]. All developmental stages (larva, nymph and male/female adults) have been found feeding on these animals [27, 31]. Moreover, one study has shown that odors related to animal health status seems to have an impact on the tick infestation rate [4]. The most common species of flea collected from hedgehogs is *Archaeopsylla erinacei* (Fig. 2) [4, 16, 18, 21, 32]. Other flea species have also been reported (*Leptopsylla segnis, Ctenocephalides canis* and *Ctencephalides felis*) [17, 18, 33, 34].

Fleas and ticks are also known to be vectors of different pathogens, thereby presenting a risk to human and animal health, causing such diseases as bartonellosis, flea-borne spotted fever rickettsiosis, plague, murine typhus for fleas and borreliosis, anaplasmosis, tick-borne spotted fever rickettsiosis, ehrlichiosis and several arboviruses for ticks [13, 16, 21, 35, 36]. Some of these flea and tick species have a weak host specificity and can bite different hosts, including wild, domestic and peri-domestic animals as well as humans; as such, they can thus participate in the exchange of microorganisms between their different hosts. [16, 18, 27, 37]. In addition, some tick species feed on a different host at each developmental stage and can thus actively participate in the spread of pathogens that could be responsible for zoonoses [38].

Many biotic factors, such as urbanization, land consolidation and large-scale mechanized agriculture, have resulted in the destruction of the hedgehog’s natural habitats, leading them to take refuge near human dwellings where they seek food and shelters in gardens and parks [2, 3]. In some countries and populations, hedgehogs can be used in traditional medicine and also eaten [3, 4]. In addition, the European hedgehog *E. europaeus* and the African pygmy hedgehog *Atelerix albiventris* are frequently taken as pets and are often transported with their parasites and pathogens from one region to another, exposing humans to several zoonotic agents, including those carried by their ectoparasites [2, 13, 18].

Two interesting reviews on hedgehog zoonoses have been published. However, the authors of these reviews mainly focused on the microorganisms detected in the hedgehogs themselves, and only partially addressed those detected in the ectoparasites collected from them by mentioning some of the pathogens associated with ticks but not those associated with fleas [2, 3].

The aim of the present review is to collate and list the information available in the literature on the microorganisms depicting a zoonotic threat that have been detected in arthropods sampled from different hedgehog species worldwide. We do not detail here the microorganisms detected in the hedgehogs themselves because this topic has been adequately covered in the two previous reviews [2, 3].

## Microorganisms detected in ticks collected from hedgehogs

### *Coxiella burnetii*

*Coxiella burnetii* is a Gram-negative obligate, intracellular bacterium [39] that is the causative agent of Q fever [20, 39]. This bacterium is widely distributed in the environment and affects different domestic and wild animal species as well as humans and arthropods [20, 27, 39], but livestock are considered to be the principal reservoir for this zoonotic disease [3, 20]. This bacterium can be transmitted to humans and animals through direct contact with infected animals or indirectly via aerosols, infected urine, feces, birth products, milk and other dairy products [3, 20, 28].

*Coxiella burnetii* has been reported in over 40 tick species collected from several animals, including hedgehogs [27]. It has been detected in *Hyalomma excavatum* collected from *Hemiechinus auritus* (the long-eared hedgehog) in Egypt (Alexandria) [28], in 3.2% (4/125) of *Ha. flava* ticks collected from *Erinaceus amurensis* (the Amur hedgehogs) captured in forests near the cesspools in Xianning City, Hubei Province, in Central China [20] and in *Ha. erinacei* sampled from *Atelerix algirus* (North African hedgehog) collected dead in Bouira Province of northern Algeria [21]. Previous studies reported *C. burnetii* in hedgehog tissues, suggesting that these animals as well as their associated ectoparasites could be considered as reservoir hosts for this pathogen [3, 20, 21]. Ticks have been suspected of playing a role in the epidemiology and transmission of *C. burnetii*. However, to our knowledge, only one experimental study has demonstrated the vectorial capacity of *Hyalomma aegyptium* ticks for *C. burnetii* [27, 40]. Moreover, ticks have been shown to transmit *C. burnetii* horizontally through their saliva and feces and also vertically through transstadial and transovarial routes [27].

### *Borrelia* spp.

*Borrelia burgdorferi* sensu lato (*B. burgdorferi* s.l.) is a spirochete bacteria and the causative agent of Lyme borreliosis, the most common vector-borne disease in Europe and USA, infecting various vertebrate reservoir hosts [3, 41–43]. Ticks, mainly the endophilic *I. hexagonus* and those of the exophilic *I. ricinus* complex, are the recognized vectors, and transmission occurs during blood meals through their infected saliva [18, 41–44]. Humans are considered as dead-end hosts and do not play a role in the epidemiology of *B. burgdorferi* s.l. [43]. The *B. burgdorferi* s.l. group contains three* Candidatus* genospecies and at least 20 validated species, including *B. burgdorferi* s.s., *B. afzelii*, *B. garinii*,* B. bavariensis* and *B. spielmanii* [41, 42, 45].

Preliminary results of a study conducted by Skuballa et al. showed that an *I. hexagonus* nymph and an *I. ricinus* female tick sampled from European hedgehogs from Karlsruhe (Germany) were infected with *B. afzelii* and *B. spielmanii* [41]. Another study conducted in Germany on *I. hexagonus* ticks collected from hedgehogs showed that two specimens were infected with *B. afzelii*, one was infected with *B. spielmanii* and one was co-infected with *B. garinii* and *B. afzelii* [42]. Furthermore, the DNA of *B. miyamotoi*, the spirochete bacterium belonging to the relapsing fever group, causing human non-specific febrile illness, and *B. bavariensis*, have been detected in both *I. hexagonus* and *I. ricinus* collected from hedgehogs [3]. These *Borrelia* species, which are either confirmed or suspected pathogens for humans, have also been previously detected in hedgehog tissue, suggesting that they may be reservoir hosts. However, further investigation is needed in order to determine the precise role that hedgehogs play in the epidemiology of these bacteria [3, 18, 41].

#### *Anaplasma phagocytophilum*

*Anaplasma phagocytophilum* is an obligate intracellular Gram-negative bacterium [31, 46]. It is an emerging tick-born zoonotic pathogen responsible for granulocytic anaplasmosis, an acute febrile disease of public health importance that affects humans, domestic and wild animals distributed worldwide [3, 12, 46]. *Ixodes* ticks, mainly *I. ricinus* in Europe, *I. scapularis* and *I. pacificus* in the Americas and *I. persulcatus* in Asia, have been reported to be the main vectors of *A. phagocytophilum*, and wild rodents are considered as natural reservoir hosts. Infection of humans is accidental, and humans are considered to be dead-end hosts [3, 12, 31, 46]*.*

*Anaplasma phagocytophilum* was detected in 15/38 (39.47%) engorged *I. hexagonus* female ticks collected from 7/14 (50%) *E. europaeus* hedgehogs from Germany [12], in 74/277 (27%) *I. hexagonus* and 6/25 (24%) *I. ricinus* ticks sampled from hedgehogs captured alive in the Netherlands, in 413/563 (73.36%) *I. ricinus* and 90/338 (26.63%) *I. hexagonus* sampled from a captive hedgehog population in Baden-Württemberg (Germany) [31] and in the same two tick species from hedgehogs in a study conducted in Belgium [3]. In addition, 4/13 (30.8%) pools of *Dermacentor nuttalli* ticks collected from hedgehogs in Hebei Province (China) were positive for *A. phagocytophilum*. *Dermacentor* ticks are unusual hosts, and their vectorial capacity should therefore be investigated [46].

Hedgehogs are often parasitized by ticks and thus can be involved in the circulation of this bacterium [3]. Indeed, *A. phagocytophilum* has also been detected in *E. europaeus* and *Erinaceus roumanicus* hedgehog blood and tissue [3, 12, 31]. The sequences obtained by Skuballa et al. from hedgehog tissue were almost identical to those they obtained from the ticks collected from the hedgehogs in a study they conducted in Germany, indicating that transmission can probably occur from one of these hosts to the other [12]. Only some of the different *A. phagocytophilum* strains have been confirmed to be human pathogens, and the reservoirs of the pathogenic strains involved in human disease remain unknown [12, 46]. Sequencing has revealed that the most frequent variant (variant ‘A’) found in the study conducted by Silaghi et al. [31] was similar to that reported previously in human, canine and equine granulocytic anaplasmosis [31]. The role of the strains found in hedgehogs and their ticks in terms of public health issues remains unclear, but the findings provided by Silaghi et al. [31] suggest they may have some involvement. Further studies are necessary to clarify this.

#### *Anaplasma marginale*

*Anaplasma marginale* is a tick-borne pathogen causing a hemolytic disease that can sometimes be severe [26]. About 20 tick species have been reported as potential vectors in which the bacteria multiply in the midgut and salivary glands. Nevertheless, *Dermacentor* spp., *Rhipicephalus annulatus* and *Rhipicephalus bursa* are considered to be the main vectors [26, 47]. Transmission can occur between some tick species through the transovarian, transstadial and intra-stadial routes, and it has been previously proven that transmission to another host is possible [26].

Using PCR and sequencing, *A. marginale* has been detected in three *Rhipicephalus turanicus* tick pools and in the blood of 2/53 (3.8%) live long-eared hedgehogs (*H. auritus*) from which the ticks were sampled. These hedgehogs were captured in urban and suburban areas around Zabol in south-eastern Iran [26]. In addition, 40 *I. hexagonus* sampled from *E. europaeus* hedgehogs in the Netherlands tested positive for this bacterium [26]. The infection in hedgehogs seems to be subject to cyclical variations, raising questions as to whether the infection is persistent, making this animal a suitable reservoir, or whether it is rapidly eliminated by the immune system [26]. Further investigations are needed to clarify the involvement of hedgehogs in the epidemiological cycle of this bacterium.

#### *Anaplasma bovis*

*Anaplasma bovis* is an Anaplasmataceae agent of bovine anaplasmosis, a tick-borne infectious disease, which parasitizes monocytes and macrophages. It causes animal body weight loss, abortions and decreased milk production, and frequently leads to death of the infected animal [48, 49]. *Anaplasma bovis* has been detected in 4% (5/125) of *Ha. flava* ticks sampled from *E. amurensis* hedgehogs captured in forests near the cesspools in Xianning City, Hubei Province, in central China [20].

#### *Ehrlichia* spp.

The DNA of two potential new uncultivated *Ehrlichia* species has been reported in ticks collected from hedgehogs. The first was phylogenetically close to *Ehrlichia ewingii* and was detected in 9.6%, (12/125) of *Ha. flava* ticks sampled from *E. amurensis* hedgehogs captured in forests near the cesspools in Xianning City, Hubei Province, in central China [20]. The second was detected in *Rh. sanguineus* s.l. and *Ha. erinacei* ticks sampled from *A. algirus* hedgehogs in Bouira Province in north-central Algeria [21]. Phylogenetic analysis revealed that the detected *Ehrlichia* spp. clustered together with another uncultured *Ehrlichia* in a clade separate from other known species [21]. These two *Ehrlichia* spp. have not been cultivated to allow formal description of the species. Their potential pathogenicity for animals and humans also remains unknown and requires investigation.

### *Rickettsia*

*Rickettsia* are obligate intracellular bacteria causing emerging and re-emerging diseases worldwide [50]. The principal vectors are hematophagous arthropods, and they are mainly associated with ticks, fleas and lice [50].

#### *Rickettsia massiliae*

Several studies conducted in Algeria reported detecting *R. massiliae* in hedgehog ticks. Indeed, this bacterium has been detected in *Rh. sanguineus* s.l. ticks sampled from hedgehogs in Algiers [51], in north-east Algeria [4] and in 40/59 (67,80%) *Rh. sanguineus* s.l. ticks sampled from dead *A. algirus* hedgehogs in the Bouira province [21]. The DNA of *R. massiliae* has also been found in 22/212 (10.38%) *Rh. sanguineus* s.l. collected from five European hedgehogs (*E. europaeus*) captured in seven districts in the north and center of Portugal [13] and in 11/12 (91.7%) *Rh. sanguineus* s.l. ticks collected from a female *E. europaeus* hedgehog that was captured in the city center of Marseille, France [16]. *Rhipicephalus sanguineus* s.l. ticks represent the main vectors of *R. massiliae*, and this bacterium has been detected in their saliva [51]. Transovarial and transstadial transmission has also been reported among the tick population, suggesting that these ticks might be potentially reservoirs [16, 52]. Cases of human infection by *R. massiliae* have been reported in the literature [13].

#### *Rickettsia conorii*

*Rickettsia conorii* is the etiological agent of Mediterranean spotted fever (MSF) rickettsiosis, an endemic zoonosis in the Mediterranean basin transmitted mainly by the brown dog tick *Rh. sanguineus* s.l. [50]. *Rickettsia conorii conorii* strain Malish has been detected in a *Rh. sanguineus* s.l. specimen collected from a hedgehog in Algiers [51].

#### Other* Rickettsia* species

Other *Rickettsia* species, including *R. japonica* and *R. raoultii*, have been detected in 4/125 (3.2%) and 3/125 (2.4%) *Ha. flava* ticks, respectively, collected from *E. amurensis* hedgehogs captured in forests near the cesspools in Xianning City, Hubei Province in central China [20]. *Rickettsia heilongjiangensis* (*R. heilongjiangensis* XY-1) was reported for the first time in 79/110 (71.8%) *Ha. flava* ticks and in 7/45 (15.6%) *E. amurensis* hedgehogs from which the positive ticks were collected in Xuyi County, southeast China [25]. *Rickettsia helvetica* has been reported in *I. hexagonus* and *I. ricinus* ticks sampled from hedgehogs in Belgium [3, 13]. The agent of Siberian tick typhus, *Rickettsia sibirica sibirica*, has been detected in ticks from hedgehogs collected in a suburb of Beijing, China, and this bacterium has been also isolated from the same hedgehog which provided the positive tick sample [4].

In the same study conducted in Xuyi County, southeast China mentioned above, a potential new species, *Candidatus* Rickettsia xuyiensis XY-2 was detected in 8/110 (7,3%) *Ha. flava* ticks collected from *E. amurensis* hedgehogs [25]. The hedgehog from which the positive ticks were collected tested also positive [25].

A potential new uncultivated *Rickettsia* species of the spotted fever group has been reported in 11.25% of *Rh. sanguineus* s.l. and in 77% of *Ha. erinacei* ticks sampled from *A. algirus* and *Paraechinus aethiopicus* hedgehogs in north-east Algeria [4], and another potential new uncultivated *Rickettsia* species close to the previous one has been detected in two *Ha. erinacei* ticks collected from *A. algirus* hedgehogs in the Bouira province in Algeria [21].

*Rickettsia* species have already been detected in hedgehogs, which suggests that they are suitable reservoir hosts. However, their precise role in the epidemiological cycle of these rickettsial agents remain poorly known [4, 13].

#### *Leptospira* spp.

*Leptospira* spp. are obligate aerobe spirochete bacteria belonging to the Leptospiraceae family and are the agent of leptospirosis, a widespread zoonotic disease. Humans can become infected after direct contact with infected urine or indirectly through a contaminated environment [3, 53].

*Leptospira* spp. was detected in two *Rh. sanguineus* s.l. ticks collected from *A. algirus* hedgehogs for the first time in Algeria. However, sequencing did not allow the identification of the species [21].

Several studies have reported *Leptospira* species in hedgehog kidneys worldwide [3]. *Leptospira interrogans* and *L. borgpetersenii* have been reported in wild hedgehogs in France, *L. interrogans* has been reported in wild *E. amurensis* (Amur hedgehogs) from China and in *A. algirus* hedgehogs from the Bouira Province, Algeria [3, 21] and *L. ballum* has also been isolated from hedgehogs [3]. These animals are strongly suspected to play a role in the epidemiology of this zoonotic disease and its transmission to humans [3]. The involvement of ticks in the cycle should be further investigated to find out whether the specimens in the study by Aouadi et al. mentioned above were positive simply because they had ingested blood from an infected host or whether they have real vectorial capacity and competence for *Leptospira* species.

**Fig. 3:**
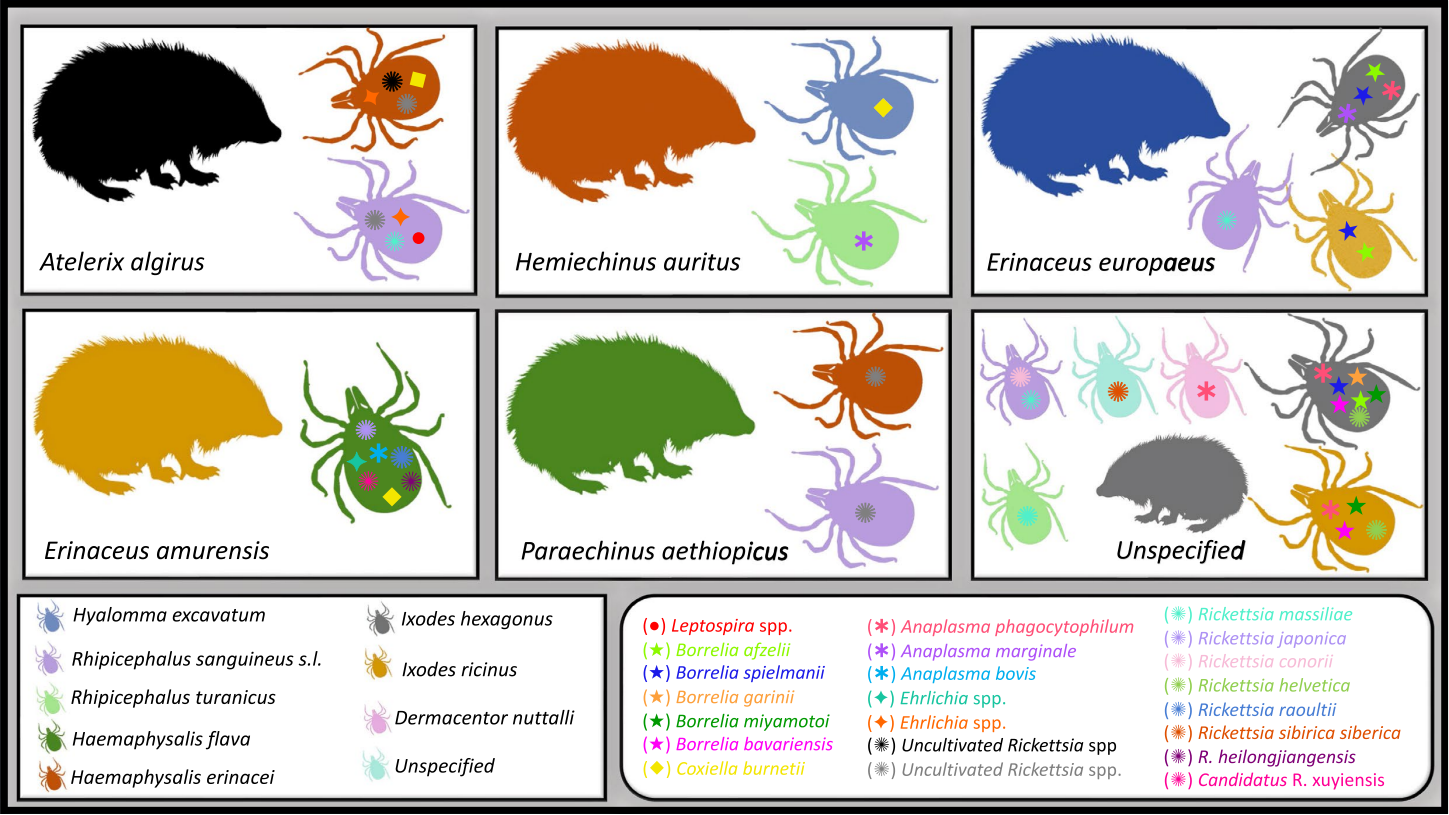
Microorganisms detected in different tick species sampled from hedgehogs worldwide

All of the microorganisms reported in the different tick species sampled from hedgehogs are summarized in Fig. 3 and in Table 1.

**Table 1:** Summary table of microorganisms detected in ticks and fleas collected from different hedgehog species worldwide

Microorganism	Arthropod	Arthropod species	hedgehog host species	Location	References
*Coxiella burnetii*	Tick	*Hyalomma excavatum*	*Hemiechinus auritus*	Alexandria, Egypt	[28]
		*Haemaphysalis flava*	*Erinaceus amurensis*	Xianning City, Hubei Province, central China	[20]
		*Haemaphysalis erinacei*	*Atelerix algirus*	Bouira province, northern Algeria	[21]
	Flea	*Archaeopsylla erinacei*	*Atelerix algirus*	Bouira province, northern Algeria	[21]
*Borrelia afzelii*	Tick	*Ixodes hexagonus*	*Erinaceus europaeus*	Karlsruhe, Germany	[41]
			Unspecified	Germany	[42]
		*Ixodes ricinus*	*Erinaceus europaeus*	Karlsruhe, Germany	[41]
*Borrelia spielmanii*	Tick	*Ixodes hexagonus*	*Erinaceus europaeus*	Karlsruhe, Germany	[41]
			Unspecified	Germany	[42]
		*Ixodes ricinus*	*Erinaceus europaeus*	Karlsruhe, Germany	[41]
*Borrelia garinii*	Tick	*Ixodes hexagonus*	Unspecified	Germany	[42]
*Borrelia miyamotoi*	Tick	*Ixodes hexagonus*	Unspecified	-	[3]
		*Ixodes ricinus*	Unspecified	-	[3]
*Borrelia bavariensis*	Tick	*Ixodes hexagonus*	Unspecified	-	[3]
		*Ixodes ricinus*	Unspecified	-	[3]
*Anaplasma phagocytophilum*	Tick	*Ixodes hexagonus*	*Erinaceus europaeus*	Germany	[12]
			unspecified	Netherlands	[31]
			Unspecified	Baden-Württemberg, Germany	[31]
			Unspecified	Belgium	[3]
		*Ixodes ricinus*	Unspecified	Netherlands	[31]
			Unspecified	Baden-Württemberg, Germany	[31]
			Unspecified	Belgium	[3]
		*Dermacentor nuttalli*	Unspecified	Hebei Province, China	[46]
*Anaplasma marginale*	Tick	*Rhipicephalus turanicus*	*Hemiechinus auritus*	Zabol, south-eastern Iran	[26]
		*Ixodes hexagonus*	*Erinaceus europaeus*	Netherlands	[26]
*Anaplasma bovis*	Tick	*Haemaphysalis flava*	*Erinaceus amurensis*	Xianning City, Hubei Province, central China	[20]
*Ehrlichia* spp. (phylogenetically close to *Ehrlichia ewingii*)	Tick	*Haemaphysalis flava*	*Erinaceus amurensis*	Xianning City, Hubei Province, central China	[20]
*Ehrlichia* spp.	Tick	*Rhipicephalus sanguineus* sensu lato	*Atelerix algirus*	Bouira province, northern Algeria	[21]
		*Haemaphysalis erinacei*	*Atelerix algirus*	Bouira province, northern Algeria	[21]
*Wolbachia* spp.	Flea	*Archaeopsylla erinacei*	*Atelerix algirus*	Bouira province, northern Algeria	[21]
			*Erinaceus europaeus*	Spain	[32]
*Rickettsia massiliae*	Tick	*Rhipicephalus sanguineus* sensu lato	Unspecified	north-east Algeria	[4]
			Unspecified	Algiers	[51]
			*Atelerix algirus*	Bouira province, northern Algeria	[21]
			*Erinaceus europaeus*	Marseille, France	[16]
			*Erinaceus europaeus*	north and centre of Portugal	[13]
		*Rhipicephalus turanicus*	Unspecified	Algiers	[51]
*Rickettsia conorii*	Tick	*Rhipicephalus sanguineus* sensu lato	Unspecified	Algiers	[51]
*Rickettsia japonica*	Tick	*Haemaphysalis flava*	*Erinaceus amurensis*	Xianning City, Hubei Province, central China	[20]
*Rickettsia raoultii*	Tick	*Haemaphysalis flava*	*Erinaceus amurensis*	Xianning City, Hubei Province, central China	[20]
*Rickettsia helvetica*	Tick	*Ixodes hexagonus*	UnspeNified	Belgium	[3]
		*Ixodes ricinus*	Unspecified	Belgium	[3]
*Rickettsia sibirica sibirica*	Tick	*unspecified*	Unspecified	suburb of Beijing	[4]
*Rickettsia heilongjiangensis*	Tick	*Haemaphysalis flava*	*Erinaceus amurensis*	Xuyi County, Southeast China	[25]
*Rickettsia felis*	Flea	*Archaeopsylla erinacei*	*Atelerix algirus*	Oran district, western Algeria	[51]
				Bouira province, northern Algeria	[21]
				north-east Algeria	[4]
				Ouled Driss, Souk Ahras, Algeria	[17]
			*Erinaceus europaeus*	Spain	[32]
				Marseille, France	[16]
			*Paraechinus aethiopicus*	North-east Algeria	[4]
		*Archaeopsylla erinacei maura*	*Erinaceus europaeus*	Mértola, southern mainland Portugal	[35]
		*Ctenocephalides felis*	*Atelerix algirus*	Ouled Driss, Souk Ahras, Algeria	[17]
*Rickettsia felis-like* organisms	Flea	*Archaeopsylla erinacei*	Unspecified	Bavaria, Germany	[58]
*Rickettsia asembonensis*	Flea	*Archaeopsylla erinacei*	*Erinaceus europaeus*	North and center Portugal	[13]
*Rickettsia typhi*	Flea	*Archaeopsylla erinacei*	*Erinaceus europaeus*	Spain	[32]
*Candidatus* Rickettsia xuyiensis	Tick	*Haemaphysalis flava*	*Erinaceus amurensis*	Xuyi County, Southeast China	[25]
Uncultivated *Rickettsia* spp.	Tick	*Rhipicephalus sanguineus* sensu lato	*Atelerix algirus*	North-east Algeria	[4]
			*Paraechinus aethiopicus*	North-east Algeria	[4]
		*Haemaphysalis erinacei*	*Atelerix algirus*	North-east Algeria	[4]
			*Paraechinus aethiopicus*	North-east Algeria	[4]
Uncultivated *Rickettsia* spp.	Tick	*Haemaphysalis erinacei*	*Atelerix algirus*	Bouira province, northern Algeria	[21]
*Leptospira* spp.	Tick	*Rhipicephalus sanguineus* sensu lato	*Atelerix algirus*	Bouira province, northern Algeria	[21]
*Bartonella henselae*	Flea	*Archaeopsylla erinacei*	*Erinaceus roumanicus*	Budapest, Hungary	[61]
*Bartonella elizabethae*	Flea	*Archaeopsylla erinacei*	Unspecified	Western Algeria	[60]
*Bartonella clarridgeiae*	Flea	*Archaeopsylla erinacei*	Unspecified	Western Algeria	[60]
Uncultivated *Bartonella* spp. (phylogenetically close to *B. clarridgeiae*)	Flea	*Archaeopsylla erinacei*	*Atelerix algirus*	Bouira province, northern Algeria	[21]
*Mycobacterium* spp.	Flea	*Archaeopsylla erinacei*	*Erinaceus europaeus*	Seville, Spain	[32]

## Microorganisms detected in fleas collected from hedgehogs

### *Rickettsia*

#### *Rickettsia felis*

**Fig. 4:**
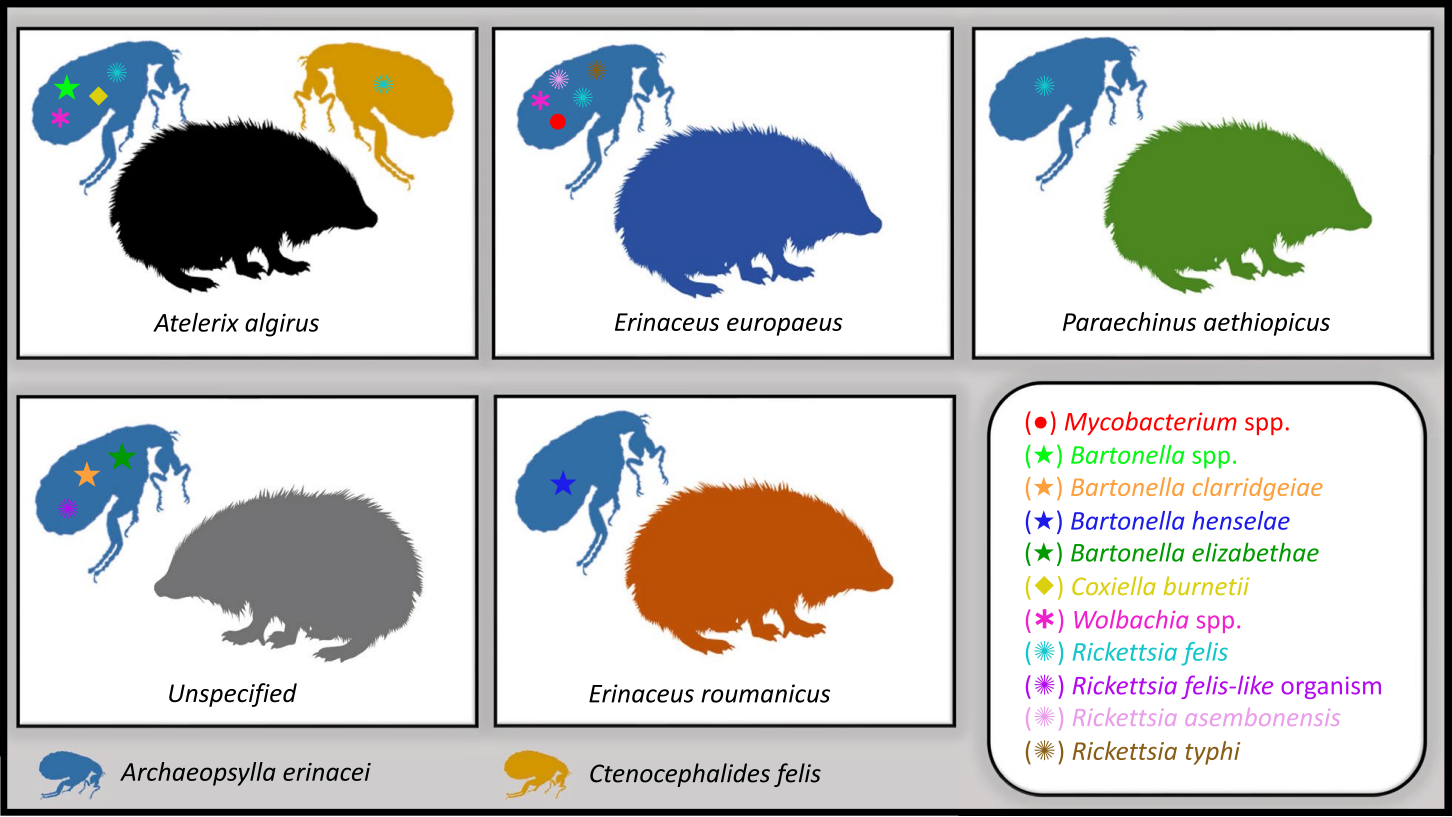
Microorganisms detected in different flea species sampled from hedgehogs worldwide

*Rickettsia felis,* the agent of flea-borne spotted fever rickettsiosis, is an emerging Gram-negative obligate intracellular bacterium reported worldwide which affects humans [54]. Although fleas, mainly cat fleas *Ctenocephalides felis,* are the recognized vectors, this bacterium has been also reported in other flea and arthropod species, such as booklice [36, 54, 55]. In addition, mosquitos have been reported to be a competent vector under experimental conditions [56] (Fig. 4)

*Rickettsia felis* have been detected in *A. erinacei* fleas collected from different hedgehog species, including *A. algirus* hedgehogs in a western region of Algeria (the Oran district) and in a north-central region of Algeria (the Bouira province) [21, 57]; in 33.3% of the fleas sampled from *E. europaeus* hedgehogs from Spain [32]; and with a high infection rate (99.2% and 95.5%) in *A. erinacei* fleas collected, respectively, from *E. europaeus* hedgehogs captured in the city center of Marseille in France, and in *A. algirus* and *P. aethiopicus* hedgehogs in Algeria [4, 16]. Furthermore, *R. felis* has been detected in *A. erinacei maura* collected from *E. europaeus* in Mértola, a southern region of mainland Portugal [35] and in *A. erinacei* and *C. felis* fleas collected from *A. algirus* hedgehogs in Cheabat El Balout (Ouled Driss, Souk Ahras) in Algeria [17]. *Rickettsia felis* transovarial transmission has been reported in fleas, suggesting that they might be potentially reservoirs. Nevertheless, the role of hedgehogs in the epidemiological cycle remains unclear, and further investigations are necessary [57].

#### *Rickettsia felis*-like organisms

A very high infection rate of a *R. felis-*like organism has been detected in *A. erinacei* fleas collected from hedgehogs in Bavaria (Germany). The authors concluded that this may be a strain variant of *R. felis* as the sequences are closely related to it and it appears to be associated with hedgehogs and their fleas [58]. Moreover, *Rickettsia asembonensis* DNA was identified in 55/117 (47%) *A. erinacei* fleas sampled from European hedgehogs (*E. europaeus*) captured in seven districts of north and center Portugal [13]. Further investigations are needed to determine the involvement of hedgehogs and their fleas in the epidemiology and circulation of *R. felis-*like organisms. Although a *R. felis-*like organism has previously been reported in a human (blood sample from a Malaysian patient), the pathogenicity of these bacteria for humans and animals remains to be determined [13, 58].

#### *Rickettsia typhi*

*Rickettsia typhi,* the etiological agent of murine typhus, has been detected in one *A. erinacei* flea sampled from an *E. europaeus* hedgehog in Spain [32]. This bacterium is usually associated with rodents and *Xenopsylla* fleas, which are considered to be the main vectors [32]. Further investigations and experimental models should be performed to find out whether this flea species could be a suitable reservoir and vector for *R. typhi*.

### *Bartonella* spp.

*Bartonella* are emerging Gram-negative fastidious oxidase-negative bacteria [59, 60]. Eleven species have been reported to be involved in various human diseases worldwide [59]. They are associated with different arthropod species, mainly fleas [35]. Transmission usually occurs through infected feces [60].

*Bartonella henselae,* the etiological agent of cat scratch disease in humans, has been detected in an *A. erinacei* flea collected on northern white-breasted hedgehogs (*E. roumanicus*) in Budapest, Hungary [61]. In addition, *Bartonella elizabethae* and *Bartonella clarridgeiae* have been detected in *A. erinacei* fleas collected from hedgehogs in Algeria (Ouled Ben Aouali and Tafraoui districts) [60]. A potential new uncultivated *Bartonella* spp. which is phylogenetically close to *B. clarridgeiae* has also been detected in the same flea species sampled from *A. algirus* hedgehogs in Algeria (Bouira province) [21].

Different *Bartonella* species have also been reported in hedgehogs. *Atelerix algirus* hedgehog spleens from Algeria have tested positive for *B. elizabethae* and *B. tribocorum* [59, 60]. *Bartonella washoensis* and *B. melophagi* have been reported in *E. europaeus* hedgehogs in urban and suburban areas of the Czech Republic [62]. Furthermore, several potential new *Bartonella* species have been reported, including clones which are phylogenetically close to *B. taylorii, B. clarridgeiae,* and *B. rochalimae* detected in the northern white-breasted hedgehog (*E. roumanicus*), the European hedgehog (*E. europaeus*), the southern white-breasted hedgehog (*E. concolor*) and the north African hedgehog (*A. algirus*) from different countries [21, 62–65]*.* The aforementioned studies highlight that *Bartonella* species have been reported in hedgehogs as well as in the fleas collected from them, suggesting that hedgehogs maybe potential reservoirs. The role of fleas as vectors of these different *Bartonella* species is deserving of further investigation through experimental models.

### *Wolbachia* spp.

*Wolbachia* are endosymbiotic bacteria of the Anaplasmataceae family which have been associated with various arthropod species [66]. *Wolbachia* spp. has been detected in *A. erinacei* fleas sampled from *A. algirus* and *E. europaeus* hedgehogs from Algeria and Spain, respectively [21, 32]. These bacteria are known to be transmitted through transovarian and transstadial routes in arthropod populations [67]. However, they have no direct impact on human and animal health.

### *Mycobacterium* spp.

Mycobacteria are Gram-positive bacilli bacteria. The diseases and symptoms that they cause in humans and animals depend on the bacterial species involved [3]. Only one published study has reported *Mycobacterium* spp. in 1/18 (5.5%) of *A. erinacei* fleas sampled from *E. europaeus* hedgehogs in Seville, Spain, but sequencing failed to identify this bacteria to the species level [32]. Different mycobacterial species, such as the causative agent of paratuberculosis in ruminants (Johne’s disease) *Mycobacterium avium paratuberculosis*, the agent of bovine tuberculosis *M. bovis* and *M. avium,* have been already reported in hedgehogs [3]. Two other studies reported *M. marinum* in both an African pygmy hedgehog (*A. albiventris*) in Japan and a European hedgehog [3]. These animals are, therefore, strongly suspected to be involved in the propagation of these bacteria. However, the involvement of fleas is still unknown, and further epidemiological investigations, together with experimental models, could help reveal whether the fleas have a vectorial capacity and competence, or whether the *A. erinacei* fleas in the study of Zurita et al. were positive only because they had ingested infected blood [3].

### *Coxiella burnetii*

As described above, *C. burnetii* is a pathogenic agent distributed worldwide [39]. Only one study has reported the presence of this bacterium in four *A. erinacei* fleas sampled from *A. algirus* hedgehogs in the Bouira province in Algeria [21]. Organs as well as ticks sampled from the same hedgehogs that provided the fleas also tested positive for this bacterium [21]. Fleas are unusual hosts for *C. burnetii*, and it can be assumed that they acquired this pathogen by co-feeding with infected ticks or by ingesting blood from an infected host [21]. Further studies are needed to determine the role of fleas in the transmission cycle of Q fever.

All of the microorganisms reported in the different flea species sampled from hedgehogs are summarized in Fig. 4 and in Table 1.

## Conclusions

This review provides information available in the literature on microorganisms detected in arthropods sampled from hedgehogs worldwide. Several microorganisms have been reported in ticks and fleas collected from these animals, including pathogenic agents which are responsible for zoonosis in animals and humans. The detection of these microorganisms in arthropods does not necessarily mean that they are vectors and that they can transmit the microorganisms to humans and animals. While the vector capacity and competence of fleas and ticks for some of these microorganisms has been proven, for others, such as *Leptospira* spp. and *Mycobacterium* spp., their detection in ticks and fleas must be viewed with some caution. They may simply have been ingested together with blood taken from an infected host. For this reason, further field epidemiological investigations, together with experimental models, are needed to clarify whether or not arthropods play a role in the maintenance and transmission of these bacteria. Until such evidence is forthcoming, these microorganisms detected in arthropods, in particular pathogenic ones, should nevertheless be added to the repertoire of microorganisms to be considered in the context of the epidemiological surveillance and diagnostic of hedgehog zoonoses.

In the present state of our knowledge, we have only a blurred picture of the role of hedgehogs as a reservoir host. Epidemiological studies on hedgehogs are difficult to conduct since they are protected animals and handling them is, therefore, highly regulated. Accordingly, their ectoparasites represent a very interesting source of information about the microorganisms circulating in these animal populations, especially vector-borne ones, without endangering them. Hedgehogs should also be dewormed to limit the risk of transmission of these pathogens to humans and other animals.

## Data Availability

All data regarding this study are included in the manuscript.
